# Highly Predictive Genetic Markers Distinguish Drug-Type from Fiber-Type *Cannabis sativa* L

**DOI:** 10.3390/plants8110496

**Published:** 2019-11-12

**Authors:** Fidelia Cascini, Alessio Farcomeni, Daniele Migliorini, Laura Baldassarri, Ilaria Boschi, Simona Martello, Stefano Amaducci, Luigi Lucini, Jamila Bernardi

**Affiliations:** 1 Institute of Public Health, Università Cattolica del Sacro Cuore, 00168 Rome, Italylaurabaldassarri72@gmail.com (L.B.); ilaria.boschi@policlinicogemelli.it (I.B.); Simona.martello@gmail.com (S.M.); 2 Department of Economics and Finance, University of Rome “Tor Vergata”, 00177 Rome, Italy; 3 Department of Computer, Control, and Management Engineering Antonio Ruberti, Sapienza University of Rome, 00185 Rome, Italy; daniele.migliorini@gmail.com; 4 Department of Sustainable Crop Production, Università Cattolica del Sacro Cuore, 29122 Piacenza, Italy; Stefano.amaducci@unicatt.it; 5 Department for Sustainable Food Process, Università Cattolica del Sacro Cuore, 29122 Piacenza, Italy; Luigi.lucini@unicatt.it

**Keywords:** SNPs, markers, *Cannabis*, *THCAS*, *CBDAS*, drug

## Abstract

Genetic markers can be used in seeds and in plants to distinguish drug-type from fiber-type *Cannabis Sativa* L. varieties even at early stages, including pre-germination when cannabinoids are not accumulated yet. With this aim, this paper reports sequencing results for *tetrahydrocannabinolic acid synthase* (*THCAS*) and *cannabidiolic acid synthase* (*CBDAS*) genes from 21 *C. sativa* L. varieties. Taking into account that *THCAS*- and *CBDAS*-derived enzymes compete for the same substrate, the novelty of this work relies in the identification of markers based on both *THCAS* and *CBDAS* rather than *THCAS* alone. Notably, in our panel, we achieved an adequate degree of discrimination (AUC 100%) between drug-type and fiber-type cannabis samples. Our sequencing approach allowed identifying multiple genetic markers (single-nucleotide polymorphisms—SNPs—and a deletion/insertion) that effectively discriminate between the two subgroups of cannabis, namely fiber type vs. drug type. We identified four functional SNPs that are likely to induce decreased *THCAS* activity in the fiber-type cannabis plants. We also report the finding on a deletion in the *CBDAS* gene sequence that produces a truncated protein, possibly resulting in loss of function of the enzyme in the drug-type varieties. Chemical analyses for the actual concentration of cannabinoids confirmed the identification of drug-type rather than fiber-type genotypes. Genetic markers permit an early identification process for forensic applications while simplifying the procedures related to detection of therapeutic or industrial hemp.

## 1. Introduction

*Cannabis sativa L.* (commonly called cannabis) is an herbaceous plant belonging to the *Cannabis* genus of the Cannabaceae family. The *Cannabis* genus includes morphologically variable varieties that can be mainly divided in two categories: Drug-type, suitable for recreational/therapeutic purposes, and fiber-type used in industrial and agronomy field. The different purposes of the cannabis genotypes depend on the ability of each variety or accession to synthesize and accumulate secondary metabolites known as cannabinoids. Cannabinoids represent a group of more than 100 natural products [1,2], of which tetrahydrocannabinol (THC) is the main (psycho) active compound. Tetrahydrocannabinolic acid (THCA) and cannabidiolic acid (CBDA) are formed from the same precursor, namely cannabigerolic acid (CBGA). The THCA synthase (THCAS) and CBDA synthase (CBDAS) are necessary to produce the metabolites THCA and CBDA [3,4,5,6,7,8].

The biosynthetic process leads to a carboxylated cannabinoid form. When cannabis inflorescences are subjected to intense heat, such as when they are smoked, cannabinoids are gradually decarboxylated to THC and cannabidiol (CBD) [9]. Thus, the phenotypic features, including the different types and contents of cannabinoids in the plant, can significantly vary among *C. sativa* varieties. It is known that some *C. sativa* L. plants lack the ability to form cannabinoids [10]. This is generally due to a knockout factor that inhibits the metabolic pathway upstream of THCAS. In these plants, neither CBD nor THC can be produced due to the absence of such upstream precursors in the biosynthetic pathway [11,12]. The sequences of THCA synthase (*THCAS*) and CBDA synthase (*CBDAS*) genes were previously characterized [6,13]. The sequence of the *CBDAS* gene has been shown to be very similar to that of the *THCAS* gene (87.9% similarity) [14]. Literature has also reported that with four single-nucleotide polymorphisms (SNPs) on *THCAS* gene, it was possible to differentiate some varieties of drug-type and non-drug cannabis plants [15]. The two cannabis types were discriminated by Kojoma and collaborators [10] studying 13 different varieties and using specific DNA polymorphisms.

Staginnus and colleagues [16] distinguished three qualitative chemotypes (chemical phenotypes) depending on the CBD/THC ratio and suggested that they differed because of polymorphisms in the THCAS sequence. De Meijer and colleagues [17] identified a locus (B) with two co-dominant alleles (BT and BD). The homozygous BT/BT genotype underlies the THC-predominant phenotype, and BD/BD underlies the CBD-predominant phenotype. The intermediate phenotype is induced by the heterozygous genotype (BT/BD). Additionally, Staginnus [16] evidenced that the qualitative chemotype was subjected to Mendelian inheritance, while the absolute quantity of THC and CBD was a quantitative trait (quantitative chemotype).

Van Bakel and colleagues [18] and, more recently, Weiblen and colleagues [19] suggested a model to explain the segregation of THCA and CBDA among marijuana and hemp cultivars. This model consists of a linked multi-locus model of inheritance in which a functional *THCAS* gene is associated with two non-functional *CBDAS* homologous genes in marijuana, and a functional *CBDAS* gene is linked to three non-functional *THCAS* homologous genes in hemp.

Considering that most of the previous activity was related to a relatively limited number of genotypes and considering that in such works only THCAS was considered, novel approaches suitable as diagnostic tests for seeds and plants are advisable. On these bases, the scope of our study was to discover highly reliable markers able to effectively discriminate fiber-type (hemp) from drug-type (marijuana) cannabis seeds and plants. We therefore considered 167 cannabis samples (both fiber-type and drug-type varieties, and both plants and seeds of the identified varieties). Seeds from each variety were grown to also include the chemical profiles of the plants at maturity related to their genotypes. Our objects considered sequencing both the *THCAS* and *CBDAS* genes (following the design of specific primers) and predicting the related proteins.

## 2. Materials and Methods

### 2.1. Experimental Cultivations and Chemical Analyses: Fiber-Type Cannabis

Ten different fiber-type (hemp) varieties of cannabis (Table S1) were chosen from among a collection of hemp at the Institute of Agronomy, DIPROVES, Università Cattolica del Sacro Cuore, Piacenza, Italy. Seeds were sown and plants were grown in an experimental field in Piacenza (North of Italy). Fifty fiber-type plants were analyzed in total (five of each variety). Inflorescences were obtained at the plant maturity stage, dried in an oven at 40 °C for 48 h, and then prepared for chemical analysis, performed according to Appendino and collaborators. [20] The samples were crushed, cleaned of seeds and secondary stems, and finely milled with a spice grinder. Next, a sub-sample (75 mg) was extracted in 15 mL methanol (reagent grade, Sigma-Aldrich, St. Louis, MO, USA) for 1 h at 50 °C in an ultrasonic bath and centrifuged at 6000× *g* for 5 min. Subsequently, an aliquot of the extract was evaporated in an oven at 50 °C for 2 h and then maintained at 120 °C for 2 h to achieve total cannabinoid decarboxylation. The samples were dissolved in the initial volume of methanol and analyzed using an in-house method based on liquid chromatography coupled to triple quadrupole tandem mass spectrometry (LC-MS/MS) via an electrospray ionization source [21]. An Agilent 1200 series liquid chromatograph and an Agilent 6410A mass spectrometer were used for this analysis. Reverse-phase chromatographic separation was achieved on a CORTECS C18 analytical column (2.7 μm, 150 mm × 3 mm inner diameter) equipped with a guard column (Waters, Milford, MA, USA) and using a binary mobile phase system (solvent A: Milli-Q water with 0.1% HCOOH; and solvent B: Methanol with 0.1% HCOOH). The gradient was increased from 75% B to 90% B in 16 min, the flow rate was 0.18 mL/min, and the column temperature was 45 °C. Cannabinoid (THC and CBD) analysis was performed under multiple reaction monitoring and positive ionization mode.

The electrospray conditions were as follows: A capillary voltage of 4000 V, vaporizer temperature of 300 °C, nitrogen flow rate of 8 L/min (18 psi), and nitrogen temperature of 300 °C. Each analyte was acquired using at least two tandem MS transitions, and the daughter ion ratio was used for confirmatory purposes, thereby achieving the required analytical specificity. Reference standards for each cannabinoid, with concentrations ranging from 0.1 to 200 mg/kg in methanol, were used as external standards for calibration and quantification purposes.

### 2.2. Experimental Cultivations and Chemical Analyses: Drug-Type Cannabis

Eleven different drug-type varieties (Table S1) were selected as suitable for indoor experimental cultivation by examining characteristic plant features such as feminization, auto-flowering, height, flowering period and THC content so that a variety of different phenotypic features were included in the examined pool of samples. For the drug-type varieties, seeds were purchased on the Internet from different online cannabis shops. Seeds purchased online were sown indoors, with one plant per pot. A total of 47 plants reached the maturity stage under controlled environmental conditions. All the experiments were performed under governmental authorizations.

Chemical analyses were performed on inflorescences and upper leaves of dried plants after indoor cultivation. Approximately 100–150 mg of each homogenized sample were solubilized in chloroform containing cholestane as an internal standard. The samples were examined by a gas chromatography-flame ionization detector (GC-FID), using a 7820A Agilent GC to identify THC and CBD and determine their percentages using a standardized analytical method. The column used was an Agilent HP-5 fused silica capillary column with a length of 30 m, an inner diameter of 0.320 mm, and a film thickness of 0.25 µm (Agilent Technologies, Santa Clara, CA, USA). The carrier gas (N_2_) flow rate was kept constant at 1 mL/min. One microliter of each sample was injected into the GC-FID using a 5:1 split injection ratio. The injector temperature was 290 °C. The column oven was programmed with an initial temperature of 200 °C for 0.5 min, followed by an increase to 260 °C at a rate of 15 °C/min, and then the temperature was maintained at 260 °C for 4 min. For the purpose of this study, the percentage values of the following cannabinoids were considered: THC, the main compound of drug-type varieties that has psychoactive effects, and CBD, the main compound of fiber-type varieties that has the same molecular precursor (CBG) as THC.

### 2.3. Isolation and Sequencing of DNA

Genetic analyses were performed on fresh leaves of each fiber-type and drug-type variety from indoor cultivation and on seeds from each subgroup. A total of 50 fiber-type and 47 drug-type plants from the experimental cultivations, as well as 50 seeds of the fiber-type varieties and 20 seeds of the drug-type varieties, were directly processed for DNA extraction, amplification and sequencing. In detail, the full-length coding sequence of *THCAS* was determined using both external and internal primers previously reported in the literature (marked by an asterisk in Table S2) [10]. The primers for *CBDAS* were designed using Primer 3plus (www.bioinformatics.nl/cgi-bin/primer3plus) to be highly specific for this gene (avoiding amplification of *THCAS*) and with the aim of gene amplification in both fiber-type and drug-type cannabis. Two primers for each gene (*THCAS* and *CBDAS*) were used to generate the full-length gene fragments, while the other primers served as internal primers in the sequencing reactions (as indicated in Table S2). A BLASTn search against GenBank (www.ncbi.gov), the specific cannabis database Comparative Genomics platform CoGe (http://genomevolution.org), and the Cannabis Genome Browser (http://genome.ccbr.utoronto.ca/) was performed for each primer. Only primers with 100% similarity to the corresponding gene were used in the following analysis.

Extraction of DNA from fresh leaves was performed using a DNeasy Plant Mini Kit (Qiagen, Hilden, Germany) following the manufacturer’s protocol; however, the protocol was adapted for the seeds (half volumes of reagents were used), which were first peeled and fragmented using a pestle and mortar. The isolated DNA was loaded in a 1% agarose gel and compared with a reference DNA sample. The amount of extracted DNA was approximately 10 ng/μL, and the quality was good for analyses of both seeds and leaves. The amplification reaction, conducted using a Qiagen Multiplex PCR Kit, was performed in a final volume of 25 μL using 10 μM primers and 5 ng of DNA. Amplification was performed with an Applied Biosystems (Foster City, CA, USA) GeneAmp PCR System 9700, and the PCR conditions were as follows: Preheating at 95 °C for 15 min followed by 30 cycles at 94 °C for 30 s, 57 °C for 90 s, and 72 °C for 90 s, and a final extension at 72° C for 10 min. The amplified products were loaded on a 2% agarose gel in 1X TBE. After being stained with ethidium bromide, the amplified products were photographed under UV light (254 nm). The products were then purified using Spin MSB PCRapace (Stratec Molecular, Berlin, Germany). Sequencing was conducted using a BigDye Terminator v3.1 Cycle Sequencing Kit (Life Technologies, Carlsbad, CA, USA) as follows: 4 μL reaction mix, 3.2 pmol of primer, and 2 μL of the purified PCR product in 15 μL total volume. The sequences were purified with a BigDye XTerminator Purification Kit and analyzed with an ABI PRISM 3130 Genetic Analyzer (Applied Biosystems).

### 2.4. Data Analysis

The sequences obtained were edited and aligned against the following reference sequences: GenBank ID KJ469374 (fiber-type *CBDAS*-cultivar Carmen) and KJ469378 (drug-type *THCAS*) from Weiblen et al. (2015). For each sample, a consensus sequence was produced by aligning all the sequences obtained, including the reverse and forward strands, to cover the entire region of the gene. The consensus sequence was generated with the SeaView platform [22]. The sequences have been submitted to GenBank with the accession numbers from MG996399 to MG996439.

The sequences were aligned and compared with previously reported *THCAS* and *CBDAS* sequences [10,13,19,23,24]. The sequences used in *THCAS* alignment were as follows: AB212836, AB212829, AB212837, and AB212830 [10], and AB057805 [13]. In addition, those used in *CBDAS* alignment were as follows: AB292682 [24], KP970864 and KP970857, and KJ469375 [19]. All sequences were aligned using the MUSCLE algorithm [25], a tool of MEGA6 software [26]. The SNPs were numbered according to the *THCAS* coding sequence of the drug-type cultivar Skunk (KJ469378) and the *CBDAS* coding sequence of the fiber-type cultivar Carmen (KJ469374). The translated protein of drug-type and fiber-type sequences of both THCAS and CBDAS were analyzed with PROVEAN [27] to predict whether the protein sequence variation affected protein function.

The fas files containing the DNA sequences were converted to the comma-separated values (csv) format using Fasta2excel online tool (http://users-birc.au.dk/biopv), and the resulting csv files were imported into R version 3.0.2. The selection of important loci was performed as follows: Each locus was treated as a categorical random variable and was used to predict the phenotype by univariate Firth’s [28] penalized-likelihood logistic regression. The resulting *p*-values were adjusted for multiplicity by Benjamini and Hochberg correction [29], which has been shown to be appropriate for use in this context of dependence of *p*-values by Farcomeni [30,31]. The list of significant loci generated after adjustment was used to generate a score, with 1 point assigned if the locus coincided with that of the consensus sequence for drug accessions, −1 point assigned if the locus coincided with that of the consensus sequence for fiber accessions, and 0 points assigned otherwise. We proceeded by comparing optimal scores based on one, two, or three up to the total number of significant loci. The optimal scores were obtained by weighting the loci to maximize the area under the receiver operating characteristics curve (AUC), under the constraint that most of the *k* weights were non-negative [32]. The parameter *k* varied from 1 to the total number of loci and differed for deletions and SNPs. Furthermore, multidimensional scaling based on Gower distances [33] was performed after grouping varieties into four groups based on THC/CBD ratio (THC/CBD up to 0.05, THC/CBD from 0.05 to 0.2, THC/CBD from 0.2 to 10, and THC/CBD more than 10).

## 3. Results

**Figure 1:**
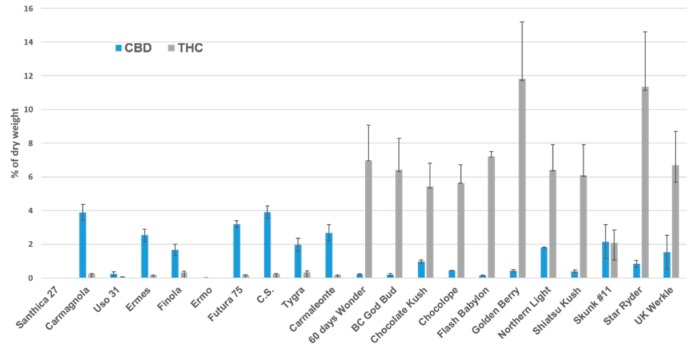
Cannabidiol (CBD) and tetrahydrocannabinol (THC) content found in drug-type and hemp varieties selected for the experiment.

Chemical and genetic analyses for two experimental cultivations of cannabis plants and genetic analyses for two pools of cannabis seeds revealed significant differences among the two varieties (fiber-type and drug-type). Chemical and genetic analyses were performed on a total of 97 plants: 47 selected among 11 different drug-type varieties and 50 selected among 10 different hemp varieties, based on the heterogeneity on CBD and/or THC content (see Figure 1). Genetic analyses only were performed on a total of 70 seeds: 20 drug-type and 50 fiber-type from the same varieties (Table S1).

### 3.1. Chemical Analysis

Results of chemical analyses performed on mature plants from experimental cultivation showed that the percentage of cannabinoids of the two subgroups of *Cannabis* was in agreement with the previously published data [10,23]. In particular, all drug-type plants showed THC concentration from 2.08% to 11.80% of dry weight, and all fiber-type plants had less than 0.31% THC. The percentage values of tetrahydrocannabinol (THC) and cannabidiol (CBD) were directly quantified using their reference standard, and the THC/CBD ratio was then calculated (Table 1).

### 3.2. Multidimensional Scaling

**Figure 2:**
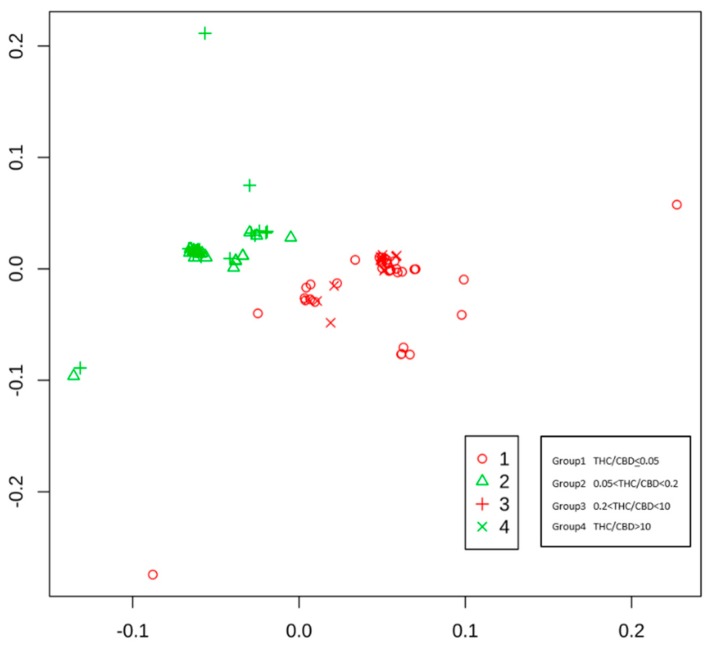
Biplot of cannabis samples with different symbols for each variety group and different colors for fiber-type and cannabis-type samples.

The sequences of all cannabis samples were different from each other. In order to assess within-variety variation, we have grouped varieties into four groups according to THC/CBD ratio (Table S3). The overall variation as assessed by average mean entropy is 0.19. The average within-group variation is 0.16, 0.07, 0.12, and 0.20 for groups 1, 2, 3, and 4, respectively. The between-group variation is 0.11. We obtained biplot based on multidimensional scaling of Gower distances after this grouping (Figure 2). It can be seen that (i) samples are non-completely overlapping, indicating that each sample has a unique DNA sequence and that (ii) fiber-type and cannabis-type samples are almost perfectly separated.

### 3.3. Discovery of Highly Predictive Markers

The comparison of sequences allowed us to identify highly reliable markers of plant type. We sequenced both the *THCAS* and the *CBDAS* genes and selected the most discriminating SNP loci (highest predictive values) from among those determined to be statistically significant, including 47 SNPs in *THCAS* and 40 in *CBDAS*. We found that some SNPs were heterozygous in drug-type samples. We decided to focus on homozygous SNPs because they are most confident and reliable for our work and future diagnostic analyses. Considering previously described four diagnostic SNPs in THCA synthase gene [15], we found that three of them (pos953, pos1035 and pos1079) were heterozygous in some drug-type samples.

**Figure 3:**
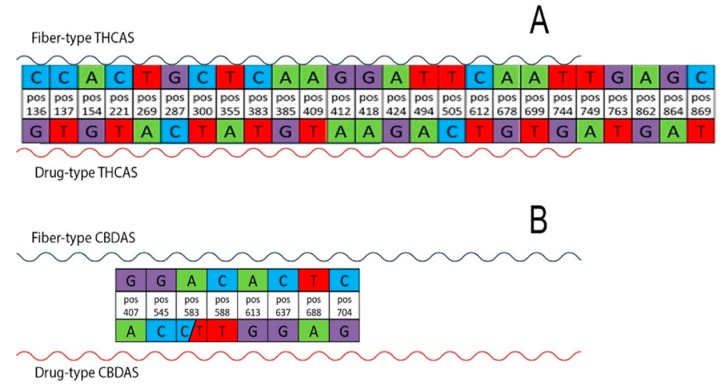
Significant single-nucleotide polymorphisms (SNPs) able to discriminate between the two cannabis subgroups of the different varieties (drug-type vs. fiber-type) are shown. Twenty-five significant SNPs are highlighted for THCAS (**A**) and eight for CBDAS (**B**). Each base change and its corresponding position (pos) in the gene are indicated.

Scores based on homozygous SNPs in the *THCAS* gene had an AUC of 100% for any of the following 25 loci: “pos136”, “pos137”, “pos154”, “pos221”, “pos269”, “pos287”, “pos300”, “pos355”, “pos383”, “pos385”, “pos409”, “pos412”, “pos418”, “pos424”, “pos494”, “pos505”, “pos612”, “pos678”, “pos699”, “pos744”, “pos749”, “pos763”, “pos862”, “pos864”, and “pos869”. Scores based on homozygous SNPs in the *CBDAS* gene were also evaluated and were found to have an AUC of 100% for any of the following eight loci: “pos407”, “pos545”, “pos583”, “pos588”, “pos613”, “pos637”, “pos688”, and “pos704”. Of these SNP-based scores, only one locus among the 33 selected loci (Figure 3A, B) was sufficient for discriminating between the two subgroups.

Furthermore, we selected other statistically significant markers, particularly the deletion/insertion polymorphism identified in CBDAS, using the sequence from a fiber-type variety (Carmen KJ469374) as the reference sequence. Specifically, we detected a deletion of four bases from positions 153–156 and an insertion of three bases at position 755 (AAC) in the drug-type varieties.

A score based on the deletion/insertion polymorphism of CBDAS was calculated by assigning 1.1 points to the deletion chosen at position 154 (drug-type varieties) and −1 point to the insertion at position 755 + 3 (drug-type varieties). The possible values of the score were thus −1, 0, 0.1, and 1.1. The AUC was 99.87% (95% CI: 99.65–100.00%) in this case, and the threshold of 0 (score > 0, indicating classification as a drug-type variety) showed 100% sensitivity (95% CI: 100.00–100.00%) and 95.56% specificity (95% CI: 88.37–100.00%). Therefore, the CBDAS deletion/insertion was also able to discriminate between the two cannabis subgroups (we also found a deletion/insertion in the *THCAS* gene that was discarded because the AUC score was 75%). The use of Benjamini and Hochberg correction [29] guaranteed that the expected proportion of falsely detected mutations was below 5%. The empirical results confirmed that all selected SNPs were highly discriminating between marijuana and hemp. Sensitivity and specificity were above 95% for several thresholds. Sensitivity analysis revealed that these outstanding results were not dependent on the scoring system used. The AUCs for these genetic markers reached 100% even when only the CBDAS deletion polymorphism was tested together with one of the 33 SNPs listed above. Any score based on mutations that perfectly separated the two subgroups of cannabis achieved an AUC of 100% and possibly 100% sensitivity and specificity. Our suggested scores also took into account the mutation prevalence in our empirical data to maximize positive and negative predicted values. The mutations most reliably separating the two subgroups were in positions 269, 494, 749, and 763 on THCAS; in positions 637 and 583 (A/T polymorphism) on CBDAS; and a CBDAS deletion of four bases in positions 153–156 (CGTA) in drug-type genotypes (a very early stop codon resulting in a truncated protein).

### 3.4. Predicted Protein Sequence of THCAS and CBDAS

**Figure 4:**
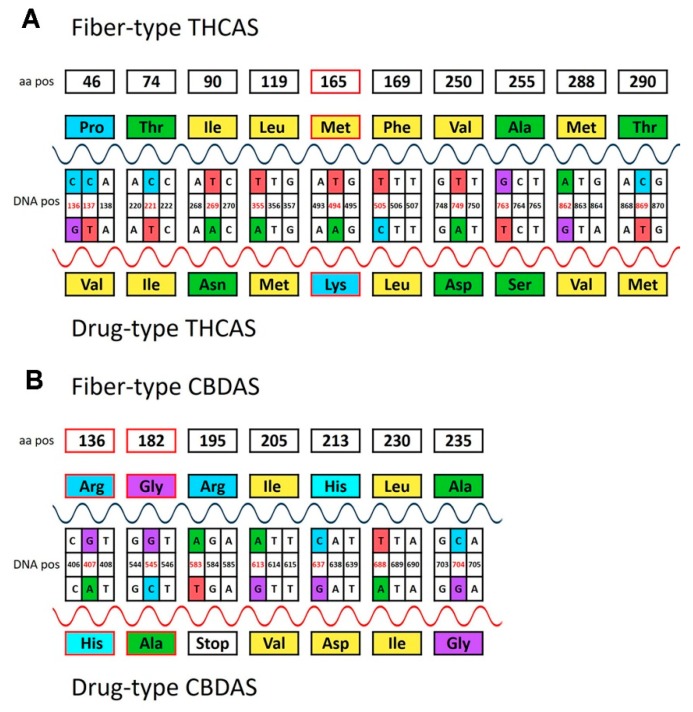
Polymorphisms of the nucleotide sequence of *tetrahydrocannabinolic acid synthase* (*THCAS*) (**A**) and *cannabidiolic acid synthase* (*CBDAS*) (**B**) causing amino acid changes in the primary structure of the protein of fiber-type and drug-type genotypes. The diagram shows the amino acid changes involving neutral (black box) and deleterious (red box) mutations of the protein sorted from PROVEAN analysis.

To test the functional meaning of the significant mutations, two artificial genes were constructed: A *THCAS* gene (containing the 25 selected SNPs of Figure 3A) and a *CBDAS* gene (containing eight SNPs plus the insertion/deletion; Figure 3B). Both genes were translated using standard genetic code from the MEGA6 platform. Polymorphisms in the primary structure of the protein were then investigated (Figure 4), and the effect of amino acid substitutions assessed according to the expected impact on secondary and tertiary structures and the mutation effect on the biological function of the protein.

With this aim, the chemical nature of the amino acids’ side chains was taken into account: changes from hydrophobic to charged structures (and vice versa) were considered of high impact, while changes from hydrophobic to polar, or from rigid/ring to fluid structures, were considered of moderate impact. The mutated *THCAS* gene gave a protein with 18 amino acid changes caused by 19 non-synonymous mutations. As expected, most of the SNPs were functional with a high non-synonymous/synonymous ratio (3.2), and 10 out of the 18 amino acid changes involved moderate to severe alteration of side chain characteristics and were thus likely to impact secondary and tertiary structures. In particular, we found that mutations in nucleotide positions 269, 494, 749, and 763 cause amino acid changes (respectively, positions 90, 165, 250, and 255) that are likely to trigger severe alterations in the protein chain. To predict whether these amino acid variations also affected protein function, we performed an in silico functional analysis with PROVEAN. This analysis revealed that the mutation at the THCA protein position 165 had a high probability to be deleterious (Figure 4A, in red). Based on this, it is reasonable to assume that the catalytic activity of THCAS is low or null in fiber-type genotypes. On the other hand, the translation of CBDAS indicated that the 4 bp deletion caused severe amino acid changes (in detail, the deletion of Leu51 and Val52) that were predicted to be deleterious for the protein function. Furthermore, a very early stop codon in position 583 of drug-type genotypes may cause a truncated protein of 195 amino acids instead of 544. The PROVEAN analysis showed that the two mutations at positions 136 (Arg/His) and 182 (Gly/Ala) were also deleterious (Figure 4B, in red). In all these cases, the corresponding CBDAS truncated and/or mutated protein would make the enzyme completely inactive.

## 4. Discussion

The aims of research on *Cannabis* varieties [1,34,35,36] have been mainly twofold: (i) to better understand the biochemical mechanisms regulating the actual chemical profile of plants (thus supporting both the toxic effects and the therapeutic applications) and (ii) to develop effective tools suitable for forensic investigations in order to counteract the illegal market and provide economic protection for the industrial cultivation of hemp. Despite efforts to establish genetic relationships, as well as to highlight genetic differences among plant varieties (with different chemical phenotypes and different psychoactive effects), these goals have remained challenging for the scientific community to date, particularly concerning the two most investigated genes in cannabis, *THCAS* and *CBDAS*. [13,19,23,24,37]).

Staginnus [16] developed a strategy to detect the BT/BT and BT/BD genotypes, independently from the developmental stage of the plant and the tissues examined, confirming the two chemotype classifications. In addition to the within-variety diversity observed by performing multidimensional scaling of Gower distances, we found a simple and reliable way for discriminating between fiber-type and drug-type samples.

Results emerging from this study showed significant genetic difference among two subgroups of *Cannabis* in two genes regulating accumulation of THC in this plant species, allowing discriminating between drug and fiber varieties. A first point of novelty in this work relates to the study of both *THCAS* and *CBDAS* genes. In fact, considering that their derived proteins compete for the same substrate, the involvement of both genes can provide a stronger and more robust discrimination between drug and fiber varieties. Nonetheless, another point of novelty related to the identification of 18 amino acid substitutions in alignment of the sequences of high-THC and low/absent-THC accessions. This information is essential to gain insights into the functionality of the enzyme. With this regard, four amino acid substitutions appeared to induce a decrease in *THCAS* activity in the fiber-type cannabis plants, and one of them was deleterious. Furthermore, the earlier stop codon at position 195 and the 4 bp deletion in the *CBDAS* sequence producing a frame-shift both cause a truncated protein and a non-functional enzyme in high-THC accessions. Assuming that the protein encoded by *THCAS* could still be active in fiber-type genotypes, the (shared) intermediate substrate cannabigerolic acid would be preferentially metabolized by the high-affinity *CBDAS*-encoded enzyme. Drug-type genotypes possess an opposite trend, having the CBDAS protein completely inactive and THCAS functional. In this condition, the substrate cannabigerolic acid could be transformed by THCAS only despite its relatively low affinity for the enzyme. Both these results were confirmed and validated at the metabolic level by the chemical analysis of cannabinoids (Table 1).

**Figure 5:**
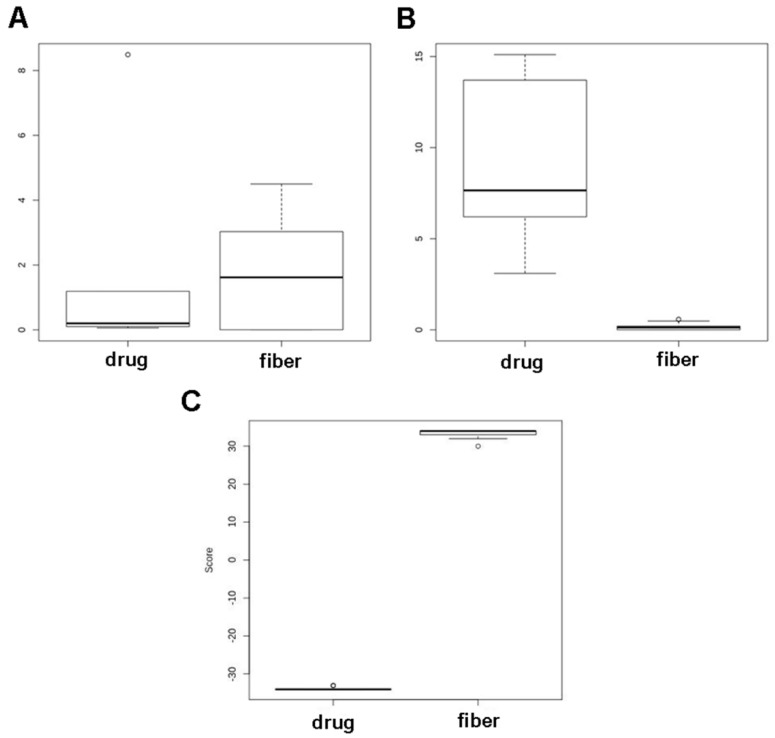
Box plot showing: CBD % (**A)** and THC % (**B**) for the drug-type and fiber-type plants based on the SNPs and deletions identified; score (**C**).

Finally, highly reliable markers were identified, including the CBDAS deletion polymorphism and the 33 identified SNPs (the eight loci in the *CBDAS* gene and 25 loci in the *THCAS* gene). We refer to the associated score as (d). The score achieved an AUC of 100%, sensitivity of 100% (95% CI: 100.00–100.00%), and specificity of 100% (95% CI: 83.33–100.00%) at the zero threshold. A boxplot of CBD and THC percentages is shown in Figure 5 (*p* < 0.001). These markers were not previously described by other authors such as Kojoma [10] and Rotherham-Harbison [15]. These markers are able to distinguish between varieties prior to the stage of plant maturity (when the synthesis and storage of cannabinoids begin). This will facilitate the early distinction of cannabis plants, as well as the selection of cannabis seeds according to their applications in the primary sector (i.e., the cultivation of hemp for textiles, cosmetics or the production of renewable energy) or pharmaceuticals (i.e., the production of cannabinoids for therapeutic use), while at the same time providing an effective tool for controlling the illicit drug market.

The future direction for this study will be to develop a rapid, highly reliable diagnostic test that maintains the lowest possible cost to expedite forensic investigations to suppress the illegal market while also providing economic protection for the industrial cultivation of hemp.

## Figures and Tables

**Table 1:** The main cannabinoid contents of the plants from the experimental cultivations collected at the mature stage. The values are expressed as a percentage of inflorescence dry weight. * SD = standars deviation.

NAME	No. of Plants	CBD	SD*	THC	SD*	THC/CBD	Group
Santhica 27	5	0.00	-	0.00	-	0.0	Fiber
Carmagnola	5	3.89	0.49	0.24	0.04	0.06	Fiber
Uso 31	5	0.24	0.13	0.03	0.02	0.12	Fiber
Ermes	5	2.53	0.37	0.15	0.03	0.06	Fiber
Finola	5	1.66	0.34	0.31	0.1	0.18	Fiber
Ermo	5	0.01	0.01	0.00	-	0.0	Fiber
Futura 75	5	3.18	0.21	0.18	0.02	0.05	Fiber
C.S.	5	3.91	0.36	0.24	0.02	0.08	Fiber
Tygra	5	1.97	0.38	0.31	0.12	0.18	Fiber
Carmaleonte	5	2.68	0.47	0.15	0.03	0.05	Fiber
60 Days Wonder	3	0.22	0.19	6.97	3.64	32.17	Drug
BC God Bud	11	0.20	0.08	6.40	3.89	32.00	Drug
Chocolate Kush	4	0.96	0.77	5.42	2.75	5.67	Drug
Chocolope	3	0.44	0.02	5.65	1.06	12.84	Drug
Flash Babylon	2	0.14	0.18	7.20	0.30	51.43	Drug
Golden Berry	2	0.42	0.35	11.80	4.30	28.10	Drug
Northern Light	4	1.81	1.84	6.40	4.90	3.53	Drug
Shiatsu Kush	8	0.40	0.19	6.10	3.39	15.44	Drug
Skunk #11	4	2.15	1.34	2.08	0.77	0.97	Drug
Star Ryder	4	0.84	1.04	11.33	3.28	13.53	Drug
UK Werkle	2	1.54	1.79	6.70	4.70	4.36	Drug

## Supplementary Materials

The following are available online at https://www.mdpi.com/2223-7747/8/11/496/s1, Table S1: Cannabis varieties analyzed. A: Fiber-type varieties, B: Drug-type varieties, Table S2: Primers used for amplification and sequencing of the *THCAS* and *CBDAS* genes. The last column shows the corresponding primer names previously used by Kojoma and collaborators [10], Table S3: Grouping of varieties based on THC/CBD ratio.
