# Supplementary material for: Highly Predictive Genetic Markers Distinguish Drug-Type from Fiber-Type Cannabis sativa L
Source: Plants (Basel). 2019 Nov 12;8(11):496. doi: 10.3390/plants8110496 (PMC6918397; doi:10.3390/plants8110496)
Supplement: Supplementary file 1 [file plants-08-00496-s001.pdf]

Table S1. *Cannabis* varieties analysed. A: Fiber-type varieties, B: Drug-type varieties

A

Fiber-type varieties	N. of plants	N. of seeds
Santhica 27	5	5
Carmagnola	5	5
Uso 31	5	5
Ermes	5	5
Finola	5	5
Ermo	5	5
Futura 75	5	5
C.S.	5	5
Tygra	5	5
Carmaleonte	5	5

B

Drug-type varieties	N. of plants	N. of seeds
60 days Wonder	3	2
BC God Bud	11	0
Chocolate Kush	4	0
Chocolope	3	2
Flash Babylon	2	2
Golden Berry	2	0
Northern Light	4	3
Shiatsu Kush	8	0
Skunk #11	4	4
Star Ryder	4	3
UK Werkle	2	4

Table S2. Primers used for amplification and sequencing of the *THCAS* and *CBDAS* genes. The last column shows the corresponding primer names previously used by Kojoma and collaborators (2006).

PRIMER NAME	SEQUENCE	APPLICATION	AMPLICON LENGTH (bp)	Tm (°C)	*from Kojoma et al. (2006)
THCAS-1-F	GACTGAAGAAAAATGAATTGCTCAGCATTTTCC	Full length/sequencing	1665	50-55	
THCAS-1-R*	TCTATTTAAAGATAATTAATGATGATGCGGTGG				primer b
THCAS-2-F	ACTGAAGAAAAATGAATTGCTCAG	Full length/sequencing	1692	60	
THCAS-2-R	ATTTAAAGATAATTAATGATGATGCG				
THCAS-3-F*	CAAACTGGTTGCTGTCCCATC	Sequencing			primer c
THCAS-3-R*	CGTCTTCTTCCCAGCTGATCT	Sequencing			primer e
THCAS-1A-F*	AGCTGGGAAGAAGACGGCTTTCTCA	Sequencing			primer d
THCAS-1A-R*	CGCCAACAGTAGGGCAATACC	Sequencing			primer f
THCAS-4-F*	AATAACTCCCATATCCAAGCA	Sequencing			primer g
THCAS-4-R*	AGGACTCGCATGATTAGTTT	Sequencing			primer h
THCAS-5-F	CTGAAGAAAAATGAATTGCTCAG	Sequencing			
THCAS-5-R	ACAATTGGTCGTGTTGAGTGTAT	Sequencing			
THCAS-6-F	TCCAAGATTGGCGTATCTCAA	Sequencing			
CBDAS-1-F	ATGAAGTGCTCAACATTCTC	Full length/sequencing	1635	53	
CBDAS-1-R	TTAATGACGATGCCGTGG				
CBDAS-2-F	TCTCCTTTTGGTTTGTTTGCAAG	Full length/sequencing	1623	60	
CBDAS-2-R	CCGTGGAAGAGGTGGGATG				
CBDAS-3-F	CATGTCTCTCATATCCAAGG	Sequencing			
CBDAS-3-R	AAACAGTAGGGCAATACCCAG	Sequencing			
CBDAS-4-F	AATCATTGTAGCATGGAAAATTAG	Sequencing			
CBDAS-4-R	AGCACCGTTCTGCCCAGCG	Sequencing			
CBDAS-5-F	GGCAGAACGGTGCTTTCAAG	Sequencing			

CBDAS-5-R	TATTTGGATTCTTGGGATCATTTA	sequencing	
CBDAS6-R	GAAGGAGTGACGATAACAAGT	sequencing	
CBDAS7-F	AAGCATCTAAACTGGATT	sequencing	
CBDAS7-R	TTAATGACGATGCCGTGGAAGAG	sequencing	

Table S3. Grouping of varieties based on THC/CBD ratio.

GROUP 1 (THC/CBD $\leq$ 0.05)	GROUP 2 (0.05 $<$ THC/CBD $<$ 0.2)	GROUP 3 (0.2 $<$ THC/CBD $<$ 10)	GROUP 4 (THC/CBD $>$ 10)
Santhica 27	Carmagnola	Chocolate Kush	60 days Wonder
Ermo	Uso 31	Northern Light	BC God Bud
Futura 75	Ermes	Skunk #11	Chocolope
Carmaleonte	Finola	UK Werkle	Flash Babylon
	C.S.		Golden Berry
	Tygra		Shiatsu Kush
			Star Ryder
